# Mutual information against correlations in binary communication channels

**DOI:** 10.1186/s12868-015-0168-0

**Published:** 2015-05-19

**Authors:** Agnieszka Pregowska, Janusz Szczepanski, Eligiusz Wajnryb

**Affiliations:** Institute of Fundamental Technological Research, Polish Academy of Sciences, Pawinskiego 5BWarsaw, PL

**Keywords:** Shannon information, Communication channel, Entropy, Mutual information, Correlation, Neuronal encoding

## Abstract

**Background:**

Explaining how the brain processing is so fast remains an open problem (van Hemmen JL, Sejnowski T., 2004). Thus, the analysis of neural transmission (Shannon CE, Weaver W., 1963) processes basically focuses on searching for effective encoding and decoding schemes. According to the Shannon fundamental theorem, mutual information plays a crucial role in characterizing the efficiency of communication channels. It is well known that this efficiency is determined by the channel capacity that is already the maximal mutual information between input and output signals. On the other hand, intuitively speaking, when input and output signals are more correlated, the transmission should be more efficient. A natural question arises about the relation between mutual information and correlation. We analyze the relation between these quantities using the binary representation of signals, which is the most common approach taken in studying neuronal processes of the brain.

**Results:**

We present binary communication channels for which mutual information and correlation coefficients behave differently both quantitatively and qualitatively. Despite this difference in behavior, we show that the noncorrelation of binary signals implies their independence, in contrast to the case for general types of signals.

**Conclusions:**

Our research shows that the mutual information cannot be replaced by sheer correlations. Our results indicate that neuronal encoding has more complicated nature which cannot be captured by straightforward correlations between input and output signals once the mutual information takes into account the structure and patterns of the signals.

## Background

Huge effort has been undertaken to analyze neuronal coding, its high efficiency and mechanisms governing them [1]. Claude Shannon published his famous paper on communication theory in 1948 [2,3]. In that paper, he formulated in a rigorous mathematical way intuitive concepts concerning the transmission of information in communication channels. The occurrences of inputs transmitted via channel and output symbols are described by random variables *X* (input) and *Y* (output). An actual important task is determination of an efficient decoding scheme; i.e., a procedure that allows a decision to be made about the sequence (message) input to the channel from the output sequence of symbols. This is the essence of the fundamental Shannon theorem, in which a crucial role is played by the capacity of the channel that is given by the maximum of mutual information over all possible probability distributions of input random variables. The theorem states that the efficiency of a channel is better when the mutual information is higher [4,5]. Analyzing a relation between data, in particular the input and response of any system, experimentalists apply the most natural tools; i.e., different types of correlations [6-14]. Correlation analysis has been used to infer the connectivity between signals. The standard correlation measure is the Pearson correlation coefficient commonly exploited in data analysis [15,16]. However, there are a number of correlation-like coefficients dedicated to specific biological and experimental phenomena [6]. Therefore, besides the Pearson correlation coefficient, in this paper, we also consider the correlation coefficient based on the spike train that is strongly related to the firing activity of neurons transmitting information. A natural question arises about the role of correlation coefficients in the description of communication channels, especially in effective decoding schemes [17,18]. Recently, interesting result has been shown [19], analytically and numerically, concerning the effects of correlations between neurons in encoding population. It turned out that decorrelation does not imply an increase in information. In [20] it was observed that the spike trains of retinal gangolin cells were indeed decorelated in comparison with the visual input. The authors conjecture that this decorrelation would enhance coding efficiency in optic nerve fibers of limited capacity. We begin a conversation about whether mutual information can be replaced in some sense by a correlation coefficient. In this paper we consider binary communication channels. It seems that the straightforward idea holds true: there is a high correlation between output and input; i.e., in the language of neuroscience, by observing a spike in the output we guess with high probability that there is also a spike in the input. This finding suggests that the mutual information and correlation coefficients behave in a similar way. In fact, we show that this is not always true and that it often happens that the mutual information and correlation coefficients behave in completely different ways.

## Methods

The communication channel is a device that acts on the input to produce the output [3,17,21]. In mathematical language, the communication channel is defined as a matrix of conditional probabilities linking the transition between input and output symbols possibly depending on the internal structure of the channel. In neuronal communication systems of the brain, information is transmitted by means of a small electric current and the timing of the action potential (mV), also known in literature as a spike train [1], plays a crucial role. Spike trains can be encoded in many ways. The most common encoding proposed in the literature is binary encoding, which is the most effective and natural method [11,22-26]. It is physically justified that spike trains as being observed, are detected with some limited time resolution *Δτ*, so that in each time slice (bin) a spike is either present or absent. If we think of a spike as representing a "1" and no spike as representing a “0”, then, if we look at some time interval of length *T*, each possible spike train is equivalent to $\frac {T}{\Delta \tau }$ digit binary number. In [26] it was shown that transient responses in auditory cortex can be described as a binary process, rather than as a highly variable Poisson process. Thus, in this paper, we analyze binary information sources and binary channels [25]. Such channels are described by a 2 × 2 matrix:

(1) $$ C= \left[ \begin{array}{ccc} p_{0|0} & p_{0|1}\\ p_{1|0} & p_{1|1} \end{array} \right],  $$

where

$$\begin{array}{@{}rcl@{}} p_{0|0}+p_{1|0}=1\quad \, \text{and}\quad p_{0|1}+p_{1|1}=1 \, \\ p_{0|0}, p_{0|1}, p_{1|0}, p_{1|1}\geq 0\ . \end{array} $$

Symbol *p*_*j*|*i*_ denotes the conditional probability of transition from state *i* to state *j*, where *i*=0,1 and *j*=0,1. Observe, that *i* and *j* are states of “different” neurons. Input symbols 0 and 1 (coming from the information source governed, in fact, by a random variable *X*) arrive with probabilities ${p_{0}^{X}}$ and ${p_{1}^{X}}$, respectively.

Having the matrix *C*, one can find a relation between these random variables; i.e., one can find by applying the standard formula $p(Y=j|X=i):=\frac {p(X=i \wedge Y=j)}{p(X=i)}$ joint probability matrix *M*(2*x*2), which in general is of the form

(2) $$ M= \left[ \begin{array}{ccc} p_{00} & p_{01} \\ p_{10} & p_{11} \end{array} \right]\,  $$

where

$$\begin{array}{@{}rcl@{}} p_{ji}=p(X=i \wedge Y=j)\quad \, \text{for}\quad i, j= 0, 1 \, \\ p_{00}+p_{01}+p_{10}+p_{11}=1 \, \\ p_{00}, p_{01}, p_{10}, p_{11}\geq 0 \,. \end{array} $$

Using this notation, the probability distributions ${p_{i}^{X}}$ and ${p_{j}^{Y}}$ of the random variables *X* and *Y* are given by

(3) $$\begin{array}{@{}rcl@{}} {p_{i}^{X}}:=p(X=i)=p_{0i}+p_{1i}\quad \, \text{for}\quad i= 0, 1 \, \\ {p_{j}^{Y}}:=p(Y=j)=p_{j0}+p_{j1} \quad \, \text{for}\quad j= 0, 1\ . \end{array} $$

The quantities ${p_{1}^{X}}$ and ${p_{1}^{Y}}$ can be interpreted as the firing rates of the input and output spike trains. We will use these probability distributions to calculate the mutual information (between input and output signals), which is expressed in terms of the entropies of the input itself, output itself and the joint probability of input and output (4). In the following, we consider two random variables *X* (input signal to the channel) and *Y* (output from the channel) both assuming only two values 0 and 1, formally both defined on the same probability space. It is well known that the correlation coefficient for any independent random variables *X* and *Y* is zero [14], but in general it is not true that *ρ*(*X*,*Y*)=0 implies independence of random variables. However, for our specific random variables *X* and *Y*, which are of binary type, most common in communication systems, we show the equivalence of independence and noncorrelation (see Appendix). The basic idea of introducing the concept of a mutual information is to determine the reduction of uncertainty (measured by entropy) of random variable *X* provided that we know the values of discrete random variable *Y*. The mutual information (*MI*) is defined as

(4) $$  MI(X,Y):=H(Y)-H(Y|X)=H(X)+H(Y)-H(X,Y) \,  $$

where *H*(*X*) is the entropy of *X*, *H*(*Y*) is the entropy of *Y*, *H*(*X*,*Y*) is the joint entropy of *X* and *Y*, and *H*(*X*|*Y*) is the conditional entropy [4,17,21,27-29]. These entropies are defined as

(5) $$\begin{array}{@{}rcl@{}} H(X):=- \Sigma_{i \in I_{s}}p(X=i)\log p(X=i) \, \\ H(Y):=- \Sigma_{j \in O_{s}}p(Y=j)\log p(Y=j) \, \end{array} $$

(6) $$ {\small{\begin{aligned} H(X,Y):=-\Sigma_{i \in I_{s}}\Sigma_{j \in O_{s}}p(X\!=i \wedge Y=j)\log p(X=i \wedge Y\!=j) \, \\ H(Y|X):= \!-\Sigma_{i \in I_{s}}p(X=i)H(Y|X=i) \, \end{aligned}}}  $$

where

(7) $$ H(Y|X=i):=-\Sigma_{j \in O_{s}}p(Y=j|X\!=i) \log p(Y\!=j|X=i) \,  $$

*I*_*s*_ and *O*_*s*_ are, in general, sets of input and output symbols, *p*(*X*=*i*) and *p*(*Y*=*j*) are probability distributions of random variables *X* and *Y*, and *p*(*X*=*i*∧*Y*=*j*) is the joint probability distribution of *X* and *Y*. Estimation of mutual information requires knowledge of the probability distributions, which may be easily estimated for two-dimensional binary distributions, but in real applications it possesses multiple problems [30]. Since, in practice, the knowledge about probability distributions is often restricted, more advanced tools must be applied, such as effective entropy estimators [24,30-33].

The relative mutual information *RMI*(*X*,*Y*) [34] between random variables *X* and Y is defined as the ratio of *MI*(*X*,*Y*) and the average of information transmitted by variables *X* and *Y*:

(8) $$ RMI(X,Y):=\frac{H(X)+H(Y)-H(X,Y)}{[H(X)+H(Y)]/2} \ .  $$

*RMI*(*X*,*Y*) measures the reduction in uncertainty of *X*, provided we have knowledge about the realization of *Y*, relative to the average uncertainty of *X* and *Y*.

It holds true that [34]

1. 0≤*RMI*(*X*,*Y*)≤1;
2. *RMI*(*X*,*Y*)=0 if and only if *X* and *Y* are independent;
3. *RMI*(*X*,*Y*)=1 if and only if there exists a deterministic relation between *X* and *Y*.

Adopting the notation (2, 3), the relative mutual information RMI can be expressed as

(9) $$ {\fontsize{8.5}{12}{\begin{aligned}  RMI(X,Y)=\frac{-\Sigma_{i=0}^{1}{p_{i}^{X}} \log {p_{i}^{X}}-\Sigma_{j=0}^{1}{p_{j}^{Y}} \log {p_{j}^{Y}}+\Sigma_{i,j=0}^{i,j=1}p_{ji} \log p_{ji}}{\left[-\Sigma_{i=0}^{1}{p_{i}^{X}} \log {p_{i}^{X}}-\Sigma_{j=0}^{1}{p_{j}^{Y}} \log {p_{j}^{Y}}\right]/2} \ . \end{aligned}}}  $$

The standard definition of the Pearson correlation coefficient *ρ*(*X*,*Y*) of random variables *X* and *Y* is

(10) $$ \begin{aligned} \rho(X,Y)&:=\frac{E[(X-EX)\cdot (Y-EY)]}{\sqrt{V(X)}\cdot \sqrt{V(Y)}}\\ &\,\,=\frac{E(X \cdot Y)-EX \cdot EY}{\sqrt{E[(X-EX)^{2}]}\sqrt{E[(Y-EY)^{2}]}} \, \end{aligned}  $$

where *E* is the average over the ensemble of elementary events, and *V*(*X*) and *V*(*Y*) are the variations of *X* and *Y*. Adopting the communication channels notation, we get

(11) $$ {\fontsize{9}{12}{\begin{aligned}  \rho(X,Y)&\,=\,\frac{p_{11}-(p_{01}+p_{11}) \cdot (p_{10}+p_{11})}{\sqrt{(p_{01}\,+\,p_{11})\,-\,(p_{01}\,+\,p_{11})^{2}}\sqrt{(p_{10}\,+\,p_{11})\,-\,(p_{10}\,+\,p_{11})^{2}}}\\ &=\frac{p_{11}-{p_{1}^{X}}{p_{1}^{Y}}}{\sqrt{{p_{0}^{X}}{p_{1}^{X}}} \cdot \sqrt{{p_{0}^{Y}}{p_{1}^{Y}}}} \ . \end{aligned}}}  $$

It follows that the Pearson correlation coefficient *ρ*(*X*,*Y*) is by no means a general measure of dependence between two random variables *X* and *Y*. *ρ*(*X*,*Y*) is connected with the linear dependence of *X* and *Y*. That is, the well-known theorem [15] states that the value of this coefficient is always between -1 and 1 and assumes -1 or 1 if and only if there exists a linear relation between *X* and *Y*.

The essence of correlation, when we describe simultaneously the input to and the output from neurons, may be expressed as the difference in the probabilities of coincident and independent spiking related to independent spiking. To realize this idea, we use a quantitative neuroscience spike-train correlation (*NSTC*) coefficient:

(12) $$ {\fontsize{9}{12}{\begin{aligned}  NSTC(X,Y)\!:=\!\frac{p_{11}\,-\,{p_{1}^{X}} \cdot {p_{1}^{Y}}}{{p_{1}^{X}} \cdot {p_{1}^{Y}}}\,=\,\frac{p_{11}\!-(p_{01}+p_{11}) \cdot (p_{10}\!+p_{11})}{(p_{01}+p_{11}) \cdot (p_{10}+p_{11})} \ . \end{aligned}}}  $$

Such a correlation coefficient with this normalization seems to be more natural than the Pearson coefficient in neuroscience. A similar idea was developed in [35] where raw-cross-correlation of simultaneous spike trains was referred to the square root of the product of firing rates. Moreover, it turns out that *NSTC* coefficient has an important property: i.e., once we know the firing rates ${p_{1}^{X}}$ and ${p_{1}^{Y}}$ of individual neurons and the coefficient, we can determine the joint probabilities of firing:

(13) $$ \begin{aligned} p_{00}=\left(1-{p_{1}^{X}}\right) \cdot \left(1-{p_{1}^{Y}}\right)+NSTC \cdot {p_{1}^{X}} \cdot {p_{1}^{Y}} \,\\ p_{01}=\left(1-{p_{1}^{Y}}\right) \cdot {p_{1}^{X}}-NSTC \cdot {p_{1}^{X}} \cdot {p_{1}^{Y}} \, \\ p_{10}={p_{1}^{Y}} \cdot \left(1-{p_{1}^{X}}\right)-NSTC \cdot {p_{1}^{X}} \cdot {p_{1}^{Y}} \, \\ p_{11}={p_{1}^{X}} \cdot {p_{1}^{Y}}+NSTC \cdot {p_{1}^{X}} \cdot {p_{1}^{Y}} \ . \end{aligned}  $$

Since *p*_11_≥0, by formula (12) we have the lower bound *NSTC*≥−1. The upper bound is unlimited for the general class (2) of joint probabilities. In the important special case when the communication channel is effective enough, i.e. *p*_11_ is large enough so the input spikes with high probability pass through the channel, one has the following practical upper bound of $NSTC<\frac {1}{p_{11}}-1$.

We present realizations of a few communication channels that show that the relative mutual information, the Pearson correlation coefficient and neuroscience spike-train correlation coefficient may behave in different ways, both qualitatively and quantitatively. Each of these realizations constitutes a family of communication channels parameterized in a continuous way by a parameter *α* from some interval. For each *α*, we propose, assuming some relation between neurons activities, the joint probability matrix of input and output signals and the information source distributions. These communication channels are determined by 2 × 2 matrixes of conditional probabilities (1). Next the joint probability is used to evaluate both the relative mutual information and correlation coefficients. Finally, we plot the values of the relative mutual information and both correlation coefficients against *α* to illustrate their different behaviors.

## Results and discussion

We start with a communication channel in which the relative mutual information monotonically increases with *α* while *NSTC* and Pearson correlation coefficients are practically constant. Moreover, *RMI* has large values which, according to the fundamental Shannon theorem, result in high transmission efficiency, while the Pearson correlation coefficient *ρ* is small. To realize these effects, we consider the situation described by the joint probability matrix (14) where the first neuron becomes more active (i.e., the probability of firing increases) with an increase in the parameter *α* while simultaneously the activity of the second neuron is unaffected by *α*. Thus, the joint probability matrix *M*(*α*) reads

(14) $$ M(\alpha)= \left[ \begin{array}{ccc} \frac{7}{15}-\alpha & \frac{1}{5}+\alpha \\ & \\ \frac{2}{15}-\alpha & \frac{1}{5}+\alpha \end{array} \right]\,.  $$

In this case, the family of the communication channels for each parameter $0<\alpha <\frac {2}{15}$ is given by the conditional probability matrix *C*(*α*):

(15) $$ C(\alpha)= \left[ \begin{array}{ccc} \frac{\frac{7}{15}-\alpha}{\frac{3}{5}-2\alpha} & \frac{\frac{1}{5}+\alpha}{\frac{2}{5}+2\alpha}\\ \frac{\frac{2}{15}-\alpha}{\frac{3}{5}-2\alpha} & \frac{\frac{1}{5}+\alpha}{\frac{2}{5}+2\alpha} \end{array} \right]\,.  $$

**Figure 1:**
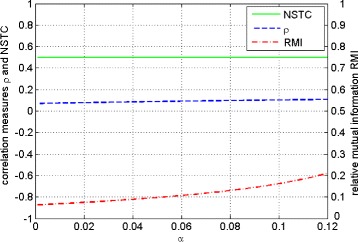
Communication channels family, Eq. (14). Course of the relative mutual information *RMI* (red dotted line), *ρ* (blue dotted line) and *NSTC* coefficient (green solid line) versus communication channels parameter *α*. The left *y*-axis corresponds to the correlation measures *ρ* and *NSTC* while the right *y*-axis corresponds to *RMI*.

We assume that the input symbols coming from an information source arrive according to the random variable *X* with probability distribution ${p_{0}^{X}}=\frac {3}{5}-2\alpha $ and ${p_{1}^{X}}=\frac {2}{5}+2\alpha $. The behaviors of *RMI*, *ρ* and the *NSTC* coefficient are presented in Figure 1.

Now consider the case for which the probability of firing of the first neuron decreases with parameter *α* while the second neuron behaves in the opposite way. The joint probability matrix *M*(*α*) we propose is

(16) $$ M(\alpha)= \left[ \begin{array}{ccc} \frac{1}{4} & \frac{7}{20}-\alpha\\ [10pt] \frac{1}{20}+2\alpha & \frac{7}{20}-\alpha \end{array} \right]\,  $$

and the information source probabilities are ${p_{0}^{X}}=\frac {3}{10}+2\alpha $ and ${p_{1}^{X}}=\frac {7}{10}-2\alpha $ for $0<\alpha <\frac {7}{20}$. Here the communication channels *C*(*α*) are of the form

(17) $$ C(\alpha)= \left[ \begin{array}{ccc} \frac{\frac{1}{4}}{\frac{3}{10}+2\alpha} & \frac{\frac{7}{20}-\alpha}{\frac{7}{10}-2\alpha}\\ [15pt] \frac{\frac{1}{20}+2\alpha}{\frac{3}{10}+2\alpha} & \frac{\frac{7}{20}-\alpha}{\frac{7}{10}-2\alpha} \end{array} \right]\ .  $$

**Figure 2:**
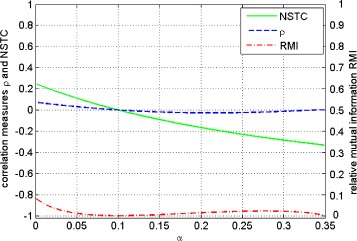
Communication channels family, Eq. (16). Course of the relative mutual information *RMI* (red dotted line), *ρ* (blue dotted line) and *NSTC* coefficient (green solid line) versus communication channels parameter *α*. The left *y*-axis corresponds to the correlation measures *ρ* and *NSTC* while the right *y*-axis corresponds to *RMI*.

For this family of communication channels, the *NSTC* coefficient strongly decreases from positive to negative values, while *ρ* and *RMI* vary non-monotonically around zero. Moreover, *ρ* exhibits one extreme and *RMI* two extremes. Additionally, for *α*=0.35, the *RMI* is close to zero while the *NSTC* coefficient is approximately -0.32 (Figure 2). We point out these values to stress that, according to the fundamental Shannon theorem, the transmission is not efficient (*RMI* is small), although at the same time, the activity of neurons described by the *NSTC* coefficient is relatively well correlated. Figure 2 shows the behaviors of *RMI*, *ρ* and the *NSTC* coefficient. Finally, we present the situation (18) in which one neuron does not change its activity with *α* and the activity of the other neuron increases with *α*. Additionally, in contrast to the first case, the second neuron changes its activity only when the first neuron is active.

(18) $$ M(\alpha)= \left[ \begin{array}{ccc} \frac{1}{10} & \frac{1}{20}-\alpha \\ [10pt] \frac{4}{5} & \frac{1}{20}+\alpha \end{array} \right]  $$

**Figure 3:**
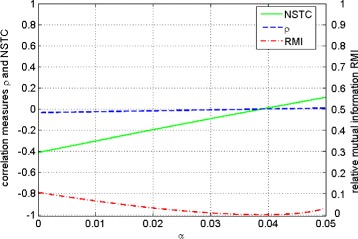
Communication channels family, Eq. (18). Course of the relative mutual information *RMI* (red dotted line), *ρ* (blue dotted line) and *NSTC* coefficient (green solid line) versus communication channels parameter *α*. The left *y*-axis corresponds to the correlation measures *ρ* and *NSTC* while the right *y*-axis corresponds to *RMI*.

In this case, the communication channel *C*(*α*) is given by

(19) $$ C(\alpha)= \left[ \begin{array}{ccc} \frac{1}{9} & \frac{\frac{1}{20}-\alpha}{\frac{1}{10}} \\ [15pt] \frac{8}{9} & \frac{\frac{1}{20}+\alpha}{\frac{1}{10}} \end{array} \right]\,  $$

and the information source probabilities are ${p_{0}^{X}}=\frac {9}{10}$ and ${p_{1}^{X}}=\frac {1}{10}$ for $0<\alpha <\frac {1}{20}$. It turns out that *NSTC* coefficient increases linearly from large negative values below -0.4 to a positive value of 0.1. Simultaneously, *ρ* is practically zero and *RMI* is small (below 0.1) but varies in a non-monotonic way having a noticeable minimum (Figure 3). Moreover, observe that for small *α* the *RMI* (equal to 0.1) is visibly larger than zero what suggests that the communication efficiency is relatively good, while at the same time the Pearson correlation coefficient *ρ* (equal to -0.03) is very close to zero, indicating that the input and output signals are almost uncorrelated (independent for binary channels). It suggests that these measures describe different qualitative properties. Figure 3 shows the behaviors of *RMI*, *ρ* and the *NSTC* coefficient.

## Conclusions

To summarize, we show that the straightforward intuitive approach of estimating the quality of communication channels according to only correlations between input and output signals is often ineffective. In other words, we refute the intuitive hypothesis which states that the more the input and output signals are correlated the more the transmission is efficient (i.e. the more effective decoding scheme can be found). This intuition could be supported by two facts:

1. for not correlated binary variables (*ρ*(*X*,*Y*)=0), (which are shown in the Appendix to be independent) one has *RMI*=0,
2. for fully correlated random variables (|*ρ*(*X*,*Y*)|=1) (which are linearly dependent) one has *RMI*=1. We introduce a few communication channels for which the correlation coefficients behave completely differently to the mutual information, which shows this intuition is erroneous.

In particular, we present the realizations of channels characterized by high mutual information for input and output signals but at the same time featuring very low correlation between these signals. On the other hand, we find channels featuring quite the opposite behavior; i.e., having very high correlation between input and output signals while the mutual information turns out to be very low. This is because the mutual information, which in fact is a crucial parameter characterizing neuronal encoding, takes into account structures (patterns) of the signals and not only their statistical properties, described by firing rates. Our research shows that neuronal encoding has a much more complicated nature that cannot be captured by straightforward correlations between input and output signals.

## Appendix

The theorem states that independence and noncorrelation are equivalent for random variables that take only two values.

### **Theorem1**.

Let *X* and *Y* be random variables, which take only two real values *a*_*x*_,*b*_*x*_ and *a*_*y*_,*b*_*y*_, respectively. Let *M* be the joint probability matrix

(20) $$ M= \left[ \begin{array}{cc} p_{00} & p_{01} \\ p_{10} & p_{11} \end{array} \right]\,  $$

where

$$ \begin{aligned} p_{00}=p(X=a_{x}\wedge Y=a_{y}) \, \\  p_{01}=p(X=b_{x}\wedge Y=a_{y}) \, \\  p_{10}=p(X=a_{x}\wedge Y=b_{y}) \, \\  p_{11}=p(X=b_{x}\wedge Y=b_{y}) \, \\  \end{aligned}  $$

and

$$ \begin{aligned} p_{00}+p_{01}+p_{10}+p_{11}=1 \, \\  p_{00}, p_{01}, p_{10}, p_{11}\geq 0 \ . \end{aligned}  $$

The probability distributions of random variables *X* and *Y* are given by

(21) $$ \begin{aligned} p_{a_{x}}^{X}:=p(X=a_{x})=p_{0i}+p_{1i} \quad \, \text{for}\quad i=0 \, \\ p_{b_{x}}^{X}:=p(X=b_{x})=p_{0i}+p_{1i} \quad \, \text{for}\quad i=1 \, \\ p_{a_{y}}^{Y}:=p(Y=a_{y})=p_{j0}+p_{j1} \quad \, \text{for}\quad j=0 \, \\ p_{b_{y}}^{Y}:=p(Y=b_{y})=p_{j0}+p_{j1} \quad \, \text{for}\quad j=1 \ . \\ \end{aligned}  $$

Adopting this notation, the condition *ρ*(*X*,*Y*)=0 implies that random variables *X* and *Y* are independent.

To prove this Theorem 1, we first show the following particular case for binary random variables.

### **Lemma1**.

Let *X*_1_ and *Y*_1_ be two random variables, which take two values 0,1 only. Let *M*_1_ be the joint probability matrix

(22) $$ M_{1}= \left[ \begin{array}{cc} p_{00} & p_{01} \\ p_{10} & p_{11} \end{array} \right]\,  $$

where

(23) $$ \begin{aligned} p_{ji}=p(X_{1}=i \wedge Y_{1}=j) \quad \, \text{for}\quad i, j=0,1 \, \\ p_{00}+p_{01}+p_{10}+p_{11}=1 \, \\ p_{00}, p_{01}, p_{10}, p_{11}\geq 0 \ . \end{aligned}  $$

The probability distributions $p_{i}^{X_{1}}$ and $p_{j}^{Y_{1}}$ of these binary random variables are given by

(24) $$ \begin{aligned} p_{i}^{X_{1}}=p(X_{1}=i)=p_{0i}+p_{1i} \quad \, \text{for}\quad i=0, 1 \, \\ p_{j}^{Y_{1}}=p(Y_{1}=j)=p_{j0}+p_{j1} \quad \, \text{for}\quad j=0, 1 \ . \end{aligned}  $$

Adopting this notation, *ρ*(*X*_1_,*Y*_1_)=0 implies that *X*_1_ and *Y*_1_ are independent.

### *Proof*.

From (11), we have

(25) $$ {\fontsize{9}{12}{\begin{aligned}  \rho(X,Y)&\,=\,\frac{p_{11}\,-\,(p_{01\!}+\!p_{11}) \cdot (p_{10\!}+\!p_{11})}{\sqrt{(p_{01}\,+\,p_{11})\,-\,(p_{01}\,+\,p_{11})^{2}}\sqrt{(p_{10}\,+\,p_{11})\,-\,(p_{10\!}+\!p_{11})^{2}}}\\ &=0 \ .  \end{aligned}}}  $$

Thus, we have *p*_11_−(*p*_01_+*p*_11_)(*p*_10_+*p*_11_)=0; i.e., *p*_11_ is factorized $p_{11}=p_{1}^{X_{1}} \cdot p_{1}^{Y_{1}}$. To prove the independence of *X*_1_ and *Y*_1_, we have to show that

$$ p_{00}=p_{0}^{X_{1}} \cdot p_{0}^{Y_{1}} \, p_{01}=p_{1}^{X_{1}} \cdot p_{0}^{Y_{1}} \, p_{10}=p_{0}^{X_{1}} \cdot p_{1}^{Y_{1}} \ .   $$

We prove the first and second equality, and the third equality can be proven analogously.

Making use of (23), we have

(26) $$ \begin{aligned} p_{01}\,+\,p_{11}\,=\,1\,-\,(p_{10}\,+\,p_{00}) \, p_{10}\,+\,p_{11}\,=\,1\,-\,(p_{01}\,+\,p_{00}) \, \end{aligned}  $$

and (25)

(27) $$ {\fontsize{9}{12}{\begin{aligned} 0\,=\,p_{11}\,-\,(p_{01}\,+\,p_{11})(p_{10}\,+\,p_{11}) \\ =p_{11}-[1-(p_{10}+p_{00})][1-(p_{01}+p_{00})] \\ \,=\,p_{11}\!-[1\!-(p_{01}\!+p_{00})\!-(p_{10}+p_{00})\,+\,(p_{10}+p_{00})(p_{01}+p_{00})] \\ =(p_{11}+p_{01}+p_{10}-1)+2p_{00}-(p_{10}+p_{00})(p_{01}+p_{00}) \\ =-p_{00}+2p_{00}-(p_{10}+p_{00})(p_{01}+p_{00}) \,. \end{aligned}}}  $$

Thus, we have

(28) $$ p_{00}=(p_{10}+p_{00})(p_{01}+p_{00})=p_{0}^{X_{1}}p_{0}^{Y_{1}} \ .  $$

Similarly, we have

(29) $$ \begin{aligned} 0=p_{11}-(p_{01}+p_{11})(p_{10}+p_{11}) \\ =p_{11}-(p_{01}+p_{11})[1-(p_{01}+p_{00})] \\ =p_{11}-[(p_{01}+p_{11})-(p_{01}+p_{11})(p_{01}+p_{00})] \\ =p_{11}-p_{01}-p_{11}+(p_{01}+p_{11})(p_{01}+p_{00}) \,. \end{aligned}  $$

Thus, we have

(30) $$ p_{01}=(p_{01}+p_{11})(p_{01}+p_{00})=p_{1}^{X_{1}} \cdot p_{0}^{Y_{1}}.  $$

To generalize this Lemma 1, we consider the following. □

### **Lemma2**.

Assuming the notation as in Lemma 1, let us define the random variables: let *X*:=(*b*_*x*_−*a*_*x*_)*X*_1_+*a*_*x*_ and *Y*:=(*b*_*y*_−*a*_*y*_)*Y*_1_+*a*_*y*_.

Under these assumptions, *ρ*(*X*,*Y*)=0 implies that *X* and *Y* are independent. In other words, divalent, uncorrelated random variables have to be independent.

### *Proof*.

The proof is straightforward and follows directly (by the linearity of the average value) from the definition of the correlation coefficient (10) and from the fact that the joint probability matrices *M*_1_ for *X*_1_ and *Y*_1_ and *M* for *X* and *Y* are formally the same. Since by Lemma 1 the random variables *X*_1_ and *Y*_1_ are independent, the random variables *X* and *Y* must also be independent.

Finally, observe that *X* takes the values *a*_*x*_,*b*_*x*_ and *Y* takes the values *a*_*y*_,*b*_*y*_ only. Therefore, Theorem 1 follows immediately from Lemma 2. □
